# Effect of Adhesive Resin as a Modeling Liquid on Elution of Resin Composite Restorations

**DOI:** 10.1155/2021/3178536

**Published:** 2021-12-28

**Authors:** Mohammadreza Maalekipour, Mehri Safari, Mehrdad Barekatain, Amirhossein Fathi

**Affiliations:** ^1^Department of Operative Dentistry, Dental School, Islamic Azad University, Isfahan (Khurasgan) Branch, Arghavaniye, Khurasgan, Isfahan, Iran; ^2^Dental Material Research Center, Department of Prosthodontics, School of Dentistry, Isfahan University of Medical Sciences, Isfahan, Iran

## Abstract

**Background:**

Adhesive resin is increasingly used as a modeling liquid for composite. Based on previous studies, elution of some components from the composite mass negatively affects the oral tissues. Since few studies have focused on the effect of adhesive resin on composite mass, this study aimed to investigate the effect of dental adhesion factors as modeling liquid on the elution of substances from composite restorations.

**Materials and Methods:**

Sixty-four composite specimens (6 × 2 mm diameter × height) were prepared in four groups (*n* = 16) by using a Teflon ring. Composite mass was incrementally applied in four layers (0.5 mm). The control group contained no material between the layers, but other groups had one of the single bond, SE bond, and wetting resin adhesives between the layers. Specimens were immersed in distilled water and methanol. The amount of released triethylene glycol dimethacrylate (TEGDMA), urethane dimethacrylate (UDMA), and camphorquinone (CQ) was monitored by gas chromatography after 24 hours and 7 days. Data were analyzed with SPSS software through Kruskal–Wallis and Mann–Whitney *U* tests (*α* = 0.05).

**Results:**

The highest rate of released TEGDMA comonomer was seen in the wetting resin group in the water medium. The highest rate of released UDMA monomer was seen in SE bond and wetting resin groups in the methanol medium after 24 hours. The highest amount of released CQ in the methanol medium was observed in the SE bond group after 7 days.

**Conclusion:**

Single bond adhesive can be used as modeling liquid since it has no significant effect on the elution of components from composite mass. Whereas, wetting resin and SE bond adhesives are not suitable to be used as modeling liquid due to the high amounts of released TEGDMA and UDMA.

## 1. Introduction

Composite resins were introduced as aesthetic restorative materials in the mid-1960s. Nowadays, they are widely used as direct and indirect restorative materials [1]. Among the most common problems of composite resins is adhesion of composite to the employed instruments, which interferes with placing and modeling composites based on the anatomical form of the tooth and consequently prolongs the restoration process [2]. Clinicians try to reduce composite adhesion by using different materials, namely, isopropyl alcohol, acetone, dentin and enamel adhesives, and composite wetting resins [3, 4].

Use of low-viscosity materials, like dental adhesives, as the composite modeler liquid, is not suggested by any manufacturer; however, it is widely proposed by the clinicians. This method reduces the surface tension of a restorative material, which consequently facilitates the placement of material into the cavity and modeling process. It may also decrease the trapping of air in the restoration body, and the subsequent porosity which occurs due to the penetration of low-viscosity resin into the bubbles [5]. To this end, dental adhesives can be applied and modeled on the composite layer before light-curing or used to impregnate the instrument, put the composite into the cavity, and then model it [2]. The recently introduced resin monomers, known as “composite wetting resin” (modeling resin), are used as lubricant to prevent composite adhesion to handheld tools and facilitate composite modeling prior to polymerization [6]. Yet, the effect of these materials on the physical and mechanical properties of composite mass is not well known.

Biocompatibility of composite resin has received growing interests in recent years. Many in vitro studies have investigated the biocompatibility and toxicity of the agents eluted from the composite mass. Their findings have revealed that some of these monomers and additive agents have estrogenic, mutagenic, teratogenic, and genotoxic effects [7–10]. Elution of methacrylate monomers of the composite mass can cause allergic reactions [11] such as asthma, allergic inflammation of the eyes and nose, and contact dermatitis [12].

Whether or not composite is a biocompatible material is still under investigation. Furthermore, since composite wetting resin is a recently introduced material, studies focusing on its effects on physical and chemical properties of composite are scare. Hence, this study was designed to particularly investigate the effect of using resin adhesive as modeler liquid between the layers of resin composites on the elution of resin composite components. The null hypothesis assumed that the use of dental adhesive as modeler liquid of resin composite would not influence the concentration of substances eluted from resin composite.

## 2. Materials and Methods

### 2.1. Specimen Preparation

For this study, we used the convenient sampling method. To calculate the sample size in each group, we used the following formula:

(1) $$\begin{array}{c} n=\frac{2{\left(z_{1-\left(\alpha /2\right)}+z_{1-\beta }\right)}^{2}sd^{2}}{d^{2}}, \end{array}$$

where

(2) $$\begin{array}{c} \alpha =0.05,\\ \beta =0.1,\\ z_{1-\left(\alpha /2\right)}=1.96,\\ z_{1-\beta }=1.28, \end{array}$$

and the value of variance and *d* according to a similar previous study (Barcellos, 2008, Effects of resinous monomers used in restorative dental modeling on the cohesive strength of composite resin) are considered to be 3.25 and 4.29, respectively. So,

(3) $$\begin{array}{c} n=\frac{2{\left(z_{1-\left(\alpha /2\right)}+z_{1-\beta }\right)}^{2}sd^{2}}{d^{2}}=\frac{2\times {\left(1.96+1.28\right)}^{2}\times 3.25}{4.29^{2}}=3.708∼4. \end{array}$$

Therefore, an in vitro experimental study was performed on 64 composite resin specimens in four groups (*n* = 16). To fabricate the specimens by using a Teflon ring (6 × 2 mm internal diameter × height), the generator was placed on a glass slab. Four layers of resin composite (Filtek, Z350 XT, 3M ESPE, St. Paul, Minnesota, USA) were incrementally applied (0.5 mm per layer). The control group contained no material between the composite layers. To prepare the specimens containing modeler liquid, after placement of the first composite increment (0.5 mm per layer), the respective adhesive resin was applied on the composite surface with a disposable brush (Microbrush International, Grafton, Wisconsin, USA). Then, a new increment of composite was placed, modeled, and coated with another layer/pellicle of the modeler liquid, until the fourth increment was placed. All the specimens were prepared and standardized by the same single operator. Accordingly, the experimental groups contained one of the single bond (fifth-generation bonding; Adper^TM^ Single Bond 2 Adhesive, 3M ESPE, St. Paul, Minnesota, USA), adhesive bottle of SE bond (sixth-generation bonding, Clearfil SE Bond, Kuraray Noritake Dental Inc., Okayama, Okayama, Japan), and wetting resin (Ultradent, South Jordan, Utah, USA) between the composite layers. The modeler liquid was directly light-activated only after applying the fourth increment. No material was applied on the surface of specimens in any group. To prevent formation of the oxygen-inhibited layer, a transparent bar was placed over all specimens, and their surface was cured for 40 seconds (LED dental curing light, Demetron 2, Kerr, Middleton, Wisconsin, USA).

### 2.2. Storage and Chemical Analysis

Each group was equally divided (*n* = 8) to be separately immersed in glass vials containing either 1.5 mL of distilled water or 1.5 mL of methanol. Each subgroup was subdivided (*n* = 4) to be incubated (Memmert GmbH + Co, KG, Schwabach, Bavaria, Germany) at 37°C for either 24 hours or 7 days. The fluid in each vial was analyzed through the gas chromatography-flame-ionization detector (FID) (Agilent Technologies, Inc., Santa Clara, California, USA), and the amount of released triethylene glycol dimethacrylate (TEGDMA), urethane dimethacrylate (UDMA), and camphorquinone (CQ) was measured and recorded.

### 2.3. Statistical Analysis

The obtained data were submitted to Levene's test of the homogeneity of the group variances test and then to the statistical analysis Kruskal–Wallis and Mann–Whitney *U* tests (*α* = 0.05).

## 3. Results

**Figure 1:**
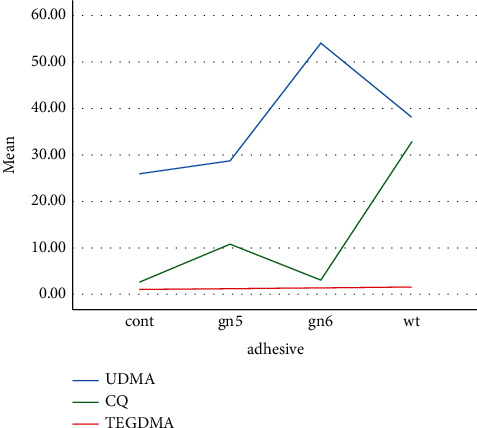
Three materials elution in different groups.

Triethylene glycol dimethacrylate (TEGDMA), urethane dimethacrylate (UDMA), and camphorquinone (CQ) were the three identified substances eluted from resin composite masses with different modeler liquids (Table 1).  Figure 1 shows the amount of eluted materials and significant differences among the groups.

### 3.1. Tests with Deuterated Methanol

**Figure 2:**
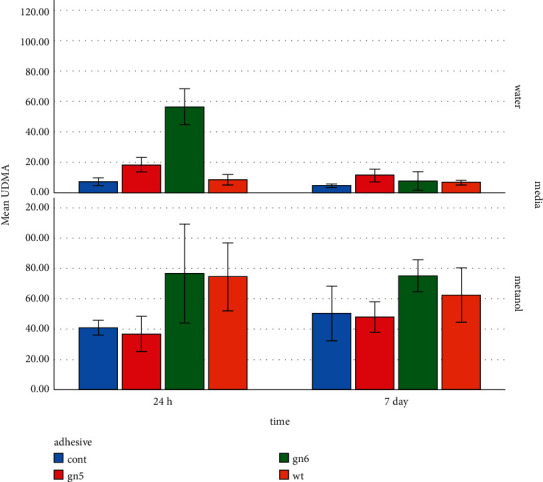
UDMA elution in different media at different times.

**Figure 3:**
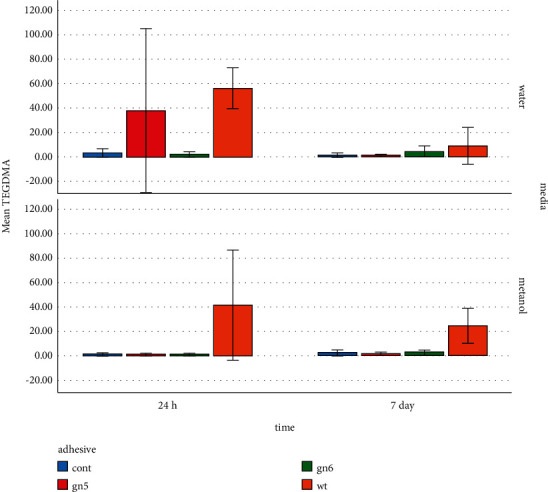
TEGDMA elution in different media at different times.

**Figure 4:**
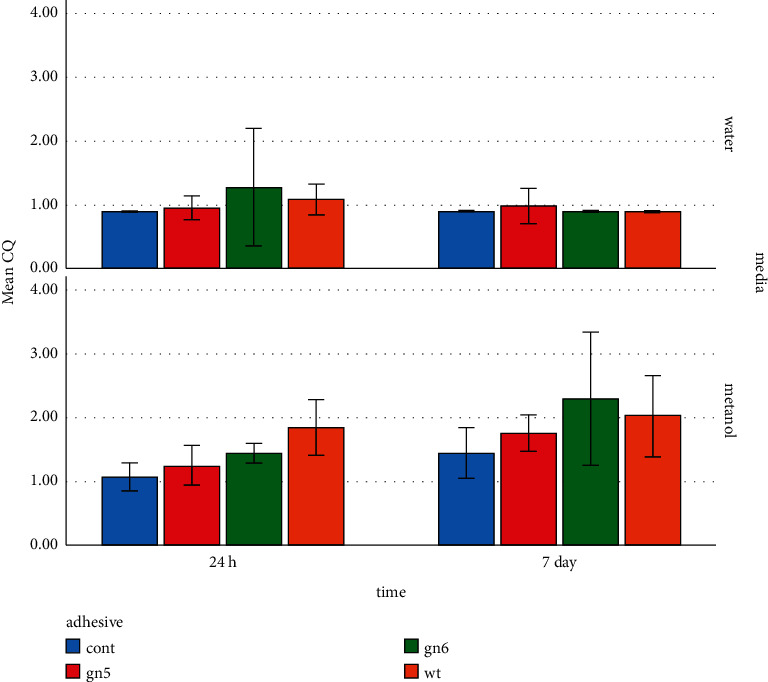
CQ elution in different media at different times.

Table 2 provides the mean (SD) concentrations of eluted constituents detected in different groups in deuterated water and deuterated methanol at different times. For the specimens immersed in methanol, the highest concentration of UDMA (76.75 mmol/l) was detected in SE bond after 24 hours (Figure 2). The highest concentration of TEGDMA (41.48 mmol/l) was measured in wetting resin after 24 hours (Figure 3). The highest concentration of CQ (2.29 mmol/l) was measured in SE bond after 7 days (Figure 4). Generally, the amount of UDMA release was higher than that of TEGDMA and CQ in all groups (Figure 1). CQ was only released in small quantities during the first 24 hours and on 7th day.

### 3.2. Tests with Deuterated Water

**Figure 5:**
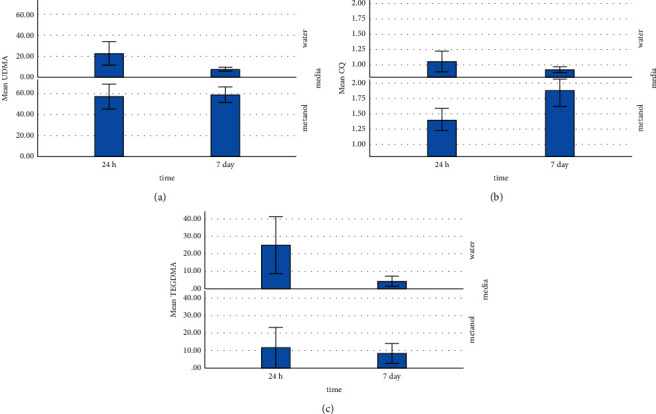
Elution rate from the three materials in different media at different times.

For the specimens immersed in deuterated water, the highest concentration of UDMA (56.50 mmol/l) was observed in the SE bond group after 24 hours (Figure 2). The highest concentration of TEGDMA (56.12 mmol/l) was found in the wetting resin group after 24 hours (Figure 3). The highest concentration of CQ (1.26 mmol/l) was detected in SE bond in deuterated water after 24 hours (Figure 4). CQ release was quite small in quantity during both the first 24 hours and 7 days. The amount of eluted liquid was higher in the specimens containing wetting resin immersed in deuterated water after 24 hours. However, after 7 days, eluted liquid was higher in the specimens immersed in methanol compared to deuterated water. The amount of liquid eluted from specimens containing wetting resin reduced in both media after 7 days; yet, it was higher than other groups (Figure 5).

### 3.3. Statistical Results

To begin with, the Levene test for homogeneity of variance was used, and a *P* value less than 0.05 was obtained. This indicates a violation of the assumption. Therefore, the nonparametric tests were used.

Following results were obtained due to our statistical analysis:

- The mean of the variables UDMA, CQ, and TEGDMA at 24 hours and 7 days did not differ significantly (at the error level 5%) as given in Table 3
- According to Table 3, the mean of the variables UDMA and CQ in water and methanol (at the error level of 5%) was significantly different. However, the mean of the variable TEGDMA in water and methanol (at the error level of 5%) was not significantly different.
- According to Table 4, the mean of the variables UDMA and TEGDMA was significantly different (at the 5% error level). In order to multiple compare two variables UDMA and TEGDMA in adhesive, we need to consider the error level to be $\alpha =\left(0.05/\left(\begin{array}{c} 4\\ 2 \end{array}\right)\right)=0.008$.
- It can be concluded according to Table 5 that at the error level of 0.008, the mean of the variable UDMA significantly differs only between the control group and gn6 group, but the mean of the variable TEGDMA significantly differs between the control and wt groups, as well as between the gn5 and wt groups, and gn6 and wt groups.

## 4. Discussion

Despite the general interest and increasing use of resin composites, concerns exist about their biochemical stability and biocompatibility [13]. Primary research focused more on improving the physical and mechanical properties of composites. In recent years, there have been growing interests in the biological safety of these materials. Previous studies on the biocompatibility of composites investigated the release of nonpolymerized residual monomers [14, 15]. The present study is the first to evaluate the effect of adhesive resins as a modeling liquid on the elution of monomers from restorative composite mass.

This study found that the amount of released TEGDMA in the wetting resin group was significantly higher than other groups during the two timespans in both water and methanol media. The amount of released TEGDMA in water was higher than that in methanol over 24 hours, which was consistent with Rothmound et al.'s study [16]. TEGDMA comonomer is reported to be more hydrophilic; thus, it is eluted in the water medium at higher quantities (more than 0.4% by weight). TEGDMA is one of the components that are highly eluted from the composite mass [17].

Ferracane [14] found a relationship between the degree of conversion and amount of released TEGDMA in the water medium, and about one-tenth of the unreacted methacrylate groups were eluted from the composite mass, which was consistent with the present study. The amount of liquid eluted from the specimens containing wetting resin reduced in both media after 7 days; yet, it was higher than other groups. It was consistent with the findings reported by Nathanson et al. [18] who stated that the maximum release of TEGDMA occurred during the first 4 minutes and then decreased. On the other hand, recent HPLC-based studies showed that composite mass releases monomers for more than 24 hours [19–21]. The reduced TEGDMA release over seven days was reported to be due to decomposition of the material into triethylene glycol and methacrylic acid [22].

In the present study, reduction of TEGDMA over 7 days of immersion in water was only observed in the wetting resin group, which could be the result of material saturation in the medium on its decomposition. The highest levels of TEGDMA were observed in the wetting resin group in methanol after 24 hours and 7 days; while, the 3 other groups showed very low amounts of released TEGDMA. The maximum rate of released TEGDMA (56.12 *μ*g/ml) was observed in the wetting resin group in the water medium after 24 hours.

TEGDMA has mutagenic activity in the laboratory environment and causes chromosomal damage [22]. Considering that EC50 (sterilizing concentration of 50% of the tested population) of TEGDMA is 1058 *μ*g/ml for human mucosal cell membrane [23], the highest level measured in this study was approximately twenty times lower than this level. TEGDMA was previously reported to exacerbate the proliferation of cariogenic microorganisms [7]. This can be significant in the wetting resin group with respect to the high release rate of this TEGDMA. Since TEGDMA is hydrophilic and easily soluble in water, it can release through the tubules shortly after the composite is placed into the pulp [24], which is seen more frequently in the remaining low-thick dentin and liner-free cavities [25]. Geurtsen [26] found that the concentration of TEGDMA-derived ED50 obtained from human pulp cells was 74.36 *μ*g/ml.

The composite specimens, used in the present study, had TEGDMA in their chemical structure. Among the groups, only wetting resin contained TEGDMA. As observed, the amount of released TEGDMA was very low and below 4 *μ*g/ml in the control group, the group in which the adhesive bottle of SE bond was used, and single bond group within the first 24 hours, which was higher than 55 *μ*g/ml in the wetting resin group. Wetting resin contains 40% filler. Its monomeric structure contains >20% Bis-GMA and >20% TEGDMA. Considering this amount of released TEGDMA, as well as the chemical properties of this material, significant elution in this group is justified by the fact that this excess amount of TEGDMA was eluted from the structure of the wetting resin layer. The material was eluted at low levels in other groups, causing no concern.

Concerning the amount of released UDMA, the present study found that the release rate was higher in the methanol medium than in distilled water, which was consistent with the results of Schuster et al.'s study [27]. Moreover, the highest amount of released UDMA was seen in the SE bond group in the water medium within 24 hours, which was significantly higher than all other 3 groups; the lowest released amount was seen in the control and wetting resin groups. There was a decrease in the release rate of UDMA in all groups after 7 days, being statistically significant in the SE bond group, indicating the degradation of UDMA in the medium. The significant reduction of released UDMA in the SE bond group after 7 days can be due to the UDMA saturation. Studies have shown that long-chain UDMA monomers can be decomposed into components such as HEMA [17, 28].

The highest release rate was seen in wetting resin and SE bond groups in methanol within 24 hours, and the lowest release occurred in single bond and control groups. The amount of released UDMA was reduced in the wetting resin and SE bond groups after 7 days; while, it increased in the control and single bond groups. It can indicate the continuous release of UDMA in unsaturated media and degradation of this material in saturated media. After 7 days, the highest amount of released UDMA was observed in the SE bond group, which was significantly different from the 3 other groups; no statistically significant difference existed among the three other groups in this regard. Overall, the highest UDMA release rate (76 *μ*g/ml) was observed in the wetting resin and SE bond groups after 24 hours in the methanol medium. Considering that EC50 (sterilizing concentration of 50% of the tested population) of UDMA for human mucosal cell membrane mucosa is 127.17 *μ*g/ml [23], the highest level recorded in the present study was approximately 1.5 times less than this level. The maximum level of UDMA in the water medium, which is similar to that of saliva, is approximately 2.5 times lower than what is considered as statistically significant.

According to Geurtsen's study [26], ED50 (sterilizing concentration of 50% of the tested population) of UDMA was 221.37 *μ*g/ml on human pulp cells. Meanwhile, Suárez et al. [29] reported that UDMA had no mutagenic effects and did not cause chromosomal damage. The only UDMA-containing material in this research was the employed composite. Hence, UDMA had released from the restorative mass, and the employed adhesives had only fluctuated the elution rate. The worst results were obtained in the SE bond group within the 24-hour period in the water medium, which showed a significant increase in the amount of UDMA elution from the composite mass, and the three other groups had a minor effect concerning the elution rate. Although UDMA has no carcinogenic and mutagenic effects by itself, it can be decomposed into components like HEMA [17, 28], which is carcinogenic and genotoxic [30].

Camphorquinone is a photoinitiator that can cause oxidative stress and DNA damage [31]. It is also known to be a potent allergen [32]. In the present study, the amount of released CQ was very small in all conditions, which was consistent with Rothmund et al.'s findings [33]. Similarly, Meng et al. [34] reported very small amounts of CQ elution, indicating that this material was polymerized almost completely. The highest and lowest amounts of CQ release in the water medium during 24 hours were observed in the SE bond and control groups, respectively; the difference was significant only between these two groups. Nevertheless, no significant difference existed among the groups after 7 days nor was any significant decrease noted in the groups over time, indicating the material release in a very low amount with a constant trend of up to a week.

Volk et al. [31] detected that 8.3 *μ*g/ml of CQ increased the intercellular reactive oxygen species (ROS) and DNA damage to human gingival fibroblasts. This amount was about thrice as much the amount obtained in methanol and 7 times more than the amount recorded in the water medium in the present research. A different study reported absence of CQ in salivary native cells. Accordingly, toxic effects are not expected in human physiological conditions [35]. CQ is a material released in small amounts, which is mainly due to the fact that CQ reacts to a large extent; therefore, no group was clearly superior to others.

Based on our findings, single bond and control groups showed the best results in terms of their noninvolvement in the elution of components from composite masses, and the wetting resin and the SE bond groups showed the worst result for TEGDMA and UDMA, respectively.

A drawback to the present study is the possibility that the use of materials has reduced the degree of resin conversion and increased the release of substances in the medium. Therefore, further investigation is recommended.

## 5. Conclusions

With respect to the present findings, it can be concluded that single bond and control groups showed the best results in terms of their noninvolvement in the elution of components from composite masses. The wetting resin and the SE bond groups showed the worst result for TEGDMA and UDMA, respectively. Although wetting resin was a lubricant in nature, it released the highest amount of TEGDMA; hence, it should be cautiously used, and its possible effects on the physical properties of materials need to be investigated.

## Figures and Tables

**Table 1:** Eluted substances identified in the study groups.

Abbreviation	Substance	Molecular formula	Molecular weight
UDMA	1,6-Bis(methacryloyloxy-2-ethoxycarbonylamino)-2,4,4-trimethylhexane	C_23_H_38_N_2_O	470
TEGDMA	Triethylene glycol dimethacrylate	C_14_H_22_O_6_	286
CQ	Camphorquinone	C_10_H_14_O_2_	166.22

**Table 2:** Mean (SD) TEGDMA, UDMA, and CQ release in the study groups.

Adhesive	Time	Media	UDMA	CQ	TEGDMA
Control (cont)	24 hours	Water	7.24 (1.57)	0.85 (0.05)	3.04 (2.15)
		Methanol	41.20 (3.10)	1.07 (0.13)	1.98 (0.10)
	7 days	Water	4.56 (0.65)	0.85 (0.05)	1.70 (0.90)
		Methanol	50.47 (11.25)	1.45 (0.25)	2.96 (1.21)

Generation 5 (gn5)	24 hours	Water	18.34 (2.98)	0.93 (0.13)	1.63 (0.31)
		Methanol	36.81 (7.44)	1.25 (0.19)	1.23 (0.07)
	7 days	Water	11.26 (2.67)	0.98 (0.17)	1.55 (0.54)
		Methanol	48.11 (6.33)	1.75 (0.18)	1.81 (0.70)

Generation 6 (gn6)	24 hours	Water	56.50 (7.41)	1.26 (0.58)	2.50 (0.89)
		Methanol	76.75 (20.45)	1.44 (0.09)	1.47 (0.28)
	7 days	Water	7.54 (3.87)	0.85 (0.05)	4.51 (2.76)
		Methanol	75.29 (6.58)	2.29 (0.65)	3.44 (0.76)

Wetting resin (wt)	24 hours	Water	8.42 (2.16)	1.09 (0.15)	56.12 (10.45)
		Methanol	74.73 (13.96)	1.84 (0.27)	41.48 (28.19)
	7 days	Water	6.77 (0.95)	.85 (0.05)	8.98 (9.45)
		Methanol	62.44(11.24)	2.02(0.40)	24.61 (8.95)

**Table 3:** Comparison of mean variables by time (24 hours and 7 days) and media (water and methanol).

	Time			Media		
	UDMA	CQ	TEGDMA	UDMA	CQ	TEGDMA
Mann–Whitney *U*	428.000	474.500	507.000	61.000	68.000	456.500
Asymp. sig. (2-tailed)	0.259	0.605	0.946	0.000^*∗*^	0.000^*∗*^	0.456

**Table 4:** Comparison of mean variables by adhesive (control, gn5, gn6, and wt).

	Adhesive		
	UDMA	CQ	TEGDMA
Kruskal–Wallis H	9.284	5.512	28.394
d*f*	3	3	3
Asymp. sig.	0.026^*∗*^	0.138	0.000^*∗*^

**Table 5:** Multiple comparison of mean variables by adhesive (control, gn5, gn6, and wt).

		UDMA	TEG
Control-gn5	Mann–Whitney *U*	103.000	86.000
	Asymp. sig.	0.346	0.113
	Exact sig.	0.361	0.119

Control-gn6	Mann–Whitney *U*	52.000	95.500
	Asymp. sig.	0.004^*∗*^	0.221
	Exact sig	0.003^*∗*^	0.224

Control-wt	Mann–Whitney *U*	82.000	11.000
	Asymp. sig.	0.083	0.000^*∗*^
	Exact sig.	0.086	0.000^*∗*^

gn5-gn6	Mann–Whitney *U*	64.000	67.000
	Asymp. sig	0.016	0.021
	Exact sig.	0.015	0.021

gn5-wt	Mann–Whitney *U*	127.000	34.000
	Asymp. sig.	0.970	0.000^*∗*^
	Exact sig	0.985	0.000^*∗*^

gn6-wt	Mann–Whitney *U*	101.000	12.500
	Asymp. sig.	0.309	0.000^*∗*^
	Exact sig.	0.323	0.000^*∗*^

## Data Availability

The data used to support the findings of this study are available from the corresponding author upon request.
